# Strategies to reduce the stigma toward people with mental disorders in Iran: stakeholders’ perspectives

**DOI:** 10.1186/s12888-016-1169-y

**Published:** 2017-01-14

**Authors:** Arsia Taghva, Zahra Farsi, Yavar Javanmard, Afsaneh Atashi, Ahmad Hajebi, Ahmad Ali Noorbala

**Affiliations:** 1 Psychiatry Department, Faculty of Medicine, AJA University of Medical Sciences, Tehran, Iran; 2 Community Health Department, Faculty of Nursing, AJA University of Medical Sciences, Kaj St., Shariati St, Tehran, Iran; 3 AJA University of Medical Sciences, Tehran, Iran; 4 Clinical Psychology Bangalore University, Bangalore, India; 5 Research Center for Addiction & Risky Behavior (ReCARB), Psychiatric Department, Iran University of Medical Sciences, Tehran, Iran; 6 Tehran University of Medical Sciences, Tehran, Iran

**Keywords:** Stigma reduction, Mental disorders, Qualitative research, Psychology, Psychiatry

## Abstract

**Background:**

Stigma affects all aspects of mental disorders, and is the most important risk factor for promoting mental health. The aim of this study was to explore strategies effective in reducing the stigma toward people with mental disorders in Iran.

**Methods:**

This qualitative study was conducted from 2013 to 2016. All participants were recruited by purposive sampling method. The majority of them were stakeholders of mental health in Iran. Data were collected through eight individual interviews, two focus groups, and six written narratives. The data were collected, coded and analyzed simultaneously. Content analysis was employed to analyze the qualitative interview data.

**Results:**

The major themes that emerged were: “Emphasis on education and changing attitudes”, “Changing the culture”, “Promoting supportive services”, “Role of various organizations and institutions”, “Integrated reform of structures and policies to improve the performance of custodians”, and “Evidence-based actions”.

**Conclusions:**

This study did not investigate the extent of stigma or its origins, rather it examines strategies appropriate for implementation in Iran. Additional studies are needed to evaluate the effectiveness of strategies for reducing the stigma attached to patients with mental disorders.

## Background

Stigma is defined as a negative stereotype and perception with prejudiced beliefs and discriminatory behavior. It creates problems for both patients and their families in livelihood activities, communication and employment, and leads to patients’ incomplete treatment [1, 2]. In other words, stigma is a pervasive term dealing with problems lack of knowledge (ignorance or misinformation), attitudes (prejudice), and behavior (discrimination) [3]. Negative attitudes toward people with mental disorders are prevalent and widespread [4]. Stigma is not only related to people with severe mental disorders [5] but people with minor psychiatric disorders may also experience stigma [6]. Contradictions and ambiguities about psychiatric patients can be considered incorrectly as cognitive deficits; thus, people who hold such views may consider them as individuals with permanent intellectual deficits [7].

Stigmatizing attitudes toward people with mental disorders have been observed in different societies and groups, including in families, co-workers [8], mental health providers [9], and students [8, 10]. Stigma may also be strengthened by patients [10, 11] who may adopt self-stigmatizing behaviors.

Several researchers have investigated the negative consequence of stigma on psychiatric patients [5, 7]. Stigma can enhance the distressing impact of mental disorders, and may lead to further complications in recovery. Such patients might suffer from many unavoidable difficulties such as discrimination, unequal rights and unemployment [12]. This is also true in finding jobs and shelter, entering higher education, obtaining insurance, and accessing to the judicial system and health support services [10]. There are two ways for rejecting psychiatric patients through prejudice and discrimination: directly (discriminative behaviors) and indirectly (marginalization) [13]. Thornicroft et al. reported exclusion and rejection of psychiatric patients in 27 countries. Rates of both anticipated and experienced discrimination among people with mental illnesses were consistently high across countries [3].

In spite of various research projects on stigma reduction programs [14, 15], few studies have examined how to overcome stigma toward people with mental disorders [10]. Thornicroft et al. found that social contact is the most effective type of intervention to improve stigma-related knowledge and attitudes in the short-term [3]. It seems that strategies of stigma reduction vary according to the contextual factors including politics, socio-economic status, culture, religion and media [16–19]. Thus, more culturally sensitive and contextual studies are needed to explore specific condition and suitable strategies in different countries. The World Health Organization (WHO) reported that stigma creates similar problems in Western and Asian countries [20]. Iran is a Middle Eastern Islamic country with a population of approximately 79 million [21] in which religious culture is dominant. Iran is a multicultural state with different ethnic communities and different languages including Persian (Mazenderani, Gilaki and Talyshi), Azerbaijani, Kurdish, Luri, Baluchi, Turkish (Qashqaies and Turkmeni) Armenian, Georgian, Assyrian, Circassian, and Arabic. Iran has undergone rapid demographic, social and economic changes [22] such as rapid population growth, urbanization and immigration [23] in the last few decades. It has faced several adversities including an 8-year Iraq-Iran war in the 1980s, and just recently severe economic sanctions and numerous natural disasters [22]. These changes have caused increase in stress and psychosocial problems [22, 23]. Noorbala et al. showed that the prevalence of mental disorders in Tehran in 1998 was 21.5%, while in 2007, it was 34.2% [23]. Sharif et al. found that the 12-month weighted prevalence of any mental disorder was 23.6% in Iranian population [22]. Jacobsson et al. examined the internalized stigma of mental illness in Sweden and Iran. They indicated that 16% of the Swedish group experienced high stigma compared to 40% of the Teheran sample [24]. Ghanean et al. demonstrated that the experience of stigma because of mental illnesses was high in Iranian population [25]. Sadighi et al. reported moderate to high levels of self-stigma in over one fifth of individuals with bipolar-I disorder in Iran [26].

Therefore, according to the above-mentioned background, the strategies of stigma reduction in this country could differ from those identified in other countries.

Knowledge about the strategies of stigma reduction can help healthcare professionals in development of supportive interventions, and giving appropriate information and advice. Therefore, considering the role of these strategies in promotion of the quality of life in mental patients, the necessity of paying due attention to this phenomenon is deeply felt in Iran. Qualitative research can play an effective role in clearing ambiguities in this regard.

The aim of this study is to explore the opinions of stakeholders of mental health about the strategies to reduce the stigma toward people with mental disorders in Iran. It is to be noted that both experienced and anticipated discriminations among people with mental disorders are also addressed in the present study.

## Methods

### Design

This qualitative study was conducted based on content analysis method [27] during 2013–2016.

### Participants

Experts of Mental Health, Social Health and Addiction (MEHSHAD) in the Ministry of Health and Medical Education (MoHME) as a major custodian of mental health in Iran assisted the researchers to select the participants. The first participant was chosen by purposive sampling method. He was the head of an insurance organization giving mental health services in some centers and a pioneer in stigma reduction in Iran. Seven participants were proposed by MoHME, and the others were recruited by the researchers. In total, 14 participants were recruited in the study (Table 1). Sampling continued until achieving theoretical saturation. Thus, various groups of participants from many sectors having continuous communication with mental disorder patients (*n* = 12), a family member of a schizophrenia patient, and a recovered patient were recruited in the study. It was important for the researchers to understand the participants’ opinions and experiences of stigmatization and discrimination and strategies for stigma reduction.

**Table 1:** Characteristics of the participants

Code	Organization	Job
P_1_	Tehran University of Medical Sciences	Professor/Psychiatrist
P_2_	Support Forum of Schizophrenia Patients (AHEBA)	Chief/Psychiatrist
P_3_	Recovered patient	Author and social activist
P_4_	Islamic Development Organization	Consulting expert
P_5_	Tehran Municipality Health Office	Expert office
P_6_	Office of Social Pathology Prevention of Welfare Organization	Expert office/Psychiatrist
P_7_	Association of Clinical Psychology	Chief
P_8_	Family member of psychiatric patient	Board member of the Support Forum of Schizophrenia Patients (AHEBA) NGO
P_9_	University of Social Welfare and Rehabilitation Sciences	Professor/Psychiatrist
P_10_	Islamic Republic of Iran Broadcasting (IRIB)	TV Channel head/Psychiatrist
P_11_	Iranian Health Education & Promotion Association	Chief
P_12_	Office of Social Pathology Prevention of Welfare Organization	Expert office/General physician
P_13_	Armed Forces Medical Services Insurance Organization	Chief/ General physician
P_14_	Health Insurance of the Ministry of Welfare, Cooperation, Labor and Social Affairs	Expert office/ General physician

*NGO* Non-Governmental Organizations

### Data collection and analysis

Data collection began with face-to-face semi-structured interviews (SSIs) with the participants. A total of eight individual SSIs were conducted with eight participants. Two stakeholders refused to participate in the study due to the lack of sufficient time. At first, the participants were interviewed by one psychologist and one psychiatrist during a 3-month period. Reasons for doing the research were explained to the participants. The SSIs were conducted in the participants’ workplaces in Persian. An interview guide including only general questions was prepared for them (Table 2). The next probing questions were asked based on the answers the participants presented. Two major questions were included in the interview guide: “*What is your idea about stigma reduction in the field of mental health?*” and *“Which organization, governmental, private, or both will support stigma reduction?”* Each interview took about one hour to complete as indicated in the interview guide.

**Table 2:** The interview guide

Part A	1) What do you know about stigma reduction in areas of mental health?	
	2) Do you have any information about the stigma reduction programs in the field of mental health?	
	If yes, please explain how (In the world/in Iran).	
	3) Are these programs effective in your view?	
	4) What is the potential benefit/detriment of these stigma reduction programs for you and your organization?	
	5) Which of the following options shows your view about the actions taken in Iran and/or in your organization on the issue of stigma reduction?	
	a) I strongly support it.	b) I somewhat support it.
	c) I do not support, but I do not oppose it.	d) I somewhat disagree with what has been done.
	e) I strongly disagree with what has been done.	f) Certain work has been done.
Part B (For those who answered “a”, “b” or “c” to question 5)	6) Which of the following three aspects do you support for stigma reduction in mental patients (Multiple-choice selection is possible)?	
	a) Personnel, operational, and educational supports	
	b) Logistic and equipment supports	
	c) Funding required supports	
	d) Others (with description):	
	7. For those who are supporting programs for stigma reduction…	
	a) When do you offer this support?	
	b) Do you show you creativity or wait for others to do it?	
	c) Do you think the financial or human resources in the country (and/or your organization) are available to support stigma reduction programs?	
	d) In your opinion, which resources can be prepared quickly for stigma reduction?	
	e) Is it a public support? Will it include all segments of the society or a particular group (Explain if necessary)?	
	f) In what circumstances do you express your support?	
	g) Will you collaborate with another person or organization for performing these actions?	
	h) Under what circumstances will you not support?	
Part C (For those who answered “e” or “f” to question 5)	8) With which of the involved aspects in stigma reduction do you disagree (Multiple-choice selection is possible)?	
	a) Personnel, operational, and educational supports	
	b) Logistic and equipment supports	
	c) Funding required supports	
	d) Others (with description):	
	9. For those who disagree with you …	
	a) In what circumstances do you show your opposition?	
	b) Do you have creativity in showing your disagreement or wait for others to do it?	
	c) Do you have any other useful alternative programs?	
	d) What resources are available for this replacement?	
	e) Is it a public program?	
	f) In what circumstances do you join to the stigma reduction programs of MoHME?	
	g) Under what circumstances do you take action to support the stigma reduction programs?	
	10) What organization or group do you think supports stigma reduction?	
	11) What are the benefits of stigma reduction for these supporters?	
	12) Which one of these supporters is actively initiative in stigma reduction?	
	13) What organization or group do you think disagrees with stigma reduction?	
	14) What are the benefits of their disagreement with stigma reduction?	
Part D: Media	15) Is the media effective in the stigma reduction of mental health problems?	
	16) What effective action can the media do in the field of mental health?	

Since the SSIs did not lead to data saturation, the researchers decided to use other methods of data collection. Due to limited time of the stakeholders and the possibility of brainstorming in focus group discussions (FGDs), two FGDs were performed in the conference hall of the MoHME in Persian. It is necessary to mention that some questions were asked in the SSIs and not in the FGDs.

Each FGD lasted for about two hours. There were 10 participants in each FGD session. Some of the same persons participated in both the SSIs and FGDs. The FGDs were managed by one of the psychiatrists of the research team, and the other researchers attended to take notes and make the necessary arrangements. The major questions of FGDs were as follows: “*What are your experiences in the field of stigma?*”, “*What are the strategies to reduce stigma*?”, and “*What can be done about stigma reduction*?” The FGDs were analyzed as heterogeneous units, and each participant's response was presented separately (e.g., P_1_-P_14_).

Personal narratives were elicited in the course of informal conversations with the participants. There were also informal conversations as separate discussions that happened after FGDs and were included in data analysis. All interviews were audio-recorded and transcribed verbatim with the permission of the participants. At the same time, the field notes recorded immediately after interviews were reviewed. In total, two FGDs, eight individual SSIs and six written narratives were collected and analyzed. Field notes and unstructured observations were other methods of data collection. Narrative is a powerful tool in the transfer or sharing of opinions and experiences. The narrative approach captures the emotion of the moment described, rendering the event active rather than passive, infused with the latent meaning being communicated by the teller [28]. Some participants contributed in up to four activities (SSI, two FGDs, an informal conversation or written narrative). It is worth mentioning that not all quotes could have come from only SSIs, FGDs or narratives. Using multiple methods for collecting data increases rigour and depth of data. The interviews were continued until achieving data saturation and emerging themes. The transcriptions were returned to the participants for correction. The data collection, coding and analysis were accomplished simultaneously by three data coders. Constant comparison was used during the whole process of analysis to distinguish differences and similarities between the initial codes. Similar codes were grouped into categories. The categories were then organized into emerging themes. The codes were revised as needed, and any concerns about the codes were discussed. All interviews were compared with each other. After review of each interview, the methods used in the process of coding were: comparing the data, asking questions, drawing diagrams, and reviewing memos. The participants provided feedback on the findings, and their comments illustrated the themes. MAXQDA software (ver.10.0 R250412) was used to help data analysis and classification. Finally, the obtained codes and findings were translated into English.

### Rigor

Guba and Lincoln (1981) outlined credibility, dependability, fitness, and conformability as the standards of rigor (trustworthiness) in qualitative research [29]. The methods performed to increase the rigor of the findings in the present study were: proper communication with the participants, numerous interviews, long-term engagement with the studied phenomenon (24 months), full immersion in the data, member checking, peer reviewing, using multiple methods for data collection, limited reviewing of the literature, accurate recording, and reporting the whole process of the research. One of the researchers with a PhD degree in nursing had already performed various qualitative studies and published some qualitative studies in the international journals. Also three researchers with MD degrees had at least a 10-years experience of treating patients with psychiatric disorders. They also taught theoretical and practical courses in the university. Two other researchers with MSc degree had the experience of giving care for patients for about 3 years.

## Results

The data analysis led to the emergence of strategies as proposed by the participants to reduce stigma in the mental disorders (Table 3).

**Table 3:** Strategies to reduce the stigma toward people with mental disorders in Iran

Themes	Sub themes
Emphasis on education and changing attitudes	Education and changing the attitudes of health care providers
	Public education
	Utilizing the potential of Islamic clergymen
Changing the culture	Establishing cultural committees, launching campaigns, and determining a support ambassador
	The role of media
	The role of books and educational materials
	Holding festivals
	The role of popular individuals
	Introducing recovered patients
	Creating a common language
Promoting supportive services	Budget and insurance coverage
	Necessity to devise appropriate tariffs for mental health services
	Consideration of the social rights of patients
Role of various organizations and institutions	Ministry of Health and Medical Education
	Municipality
	Islamic Development Organization
	Other organizations
Integrated reform of structures and policies	Establishing committee and secretariat
	Delimiting the disciplines and preventing the involvement of non-experts
	Integration of psychiatric wards in the general hospitals
	Emphasis on having systematic and massive programs
Evidence-based actions	Research actions
	Using successful projects as a pattern

*NGO* Non-Governmental Organizations

### Emphasis on education and changing attitudes

Emphasis on education and changing attitudes is a crucial strategy to reduce stigma and has priority over budget allocation.

#### Education and changing the attitudes of healthcare providers

The majority of the participants believed that physicians play an important role in introducing psychiatry to the patients and changing their attitudes towards it. It seems that attitude modification should be included in the curricula of medical students. The participants believed that medical students achieve their information about mental patients through non-scientific literature and contacting with patients. Medical students should attend the psychiatric wards under the precise supervision of the psychiatrists and psychologists. Also it is necessary to modify the attitudes of other specialists and non-specialists. One participant said:

*“I’ve seen that pharmacists or pharmacy technicians scare the patients and ask them to stop taking their medications.… So we have to educate this group of people as well.”* (P_3_WN, SSI)

The responsibility of psychiatrists and psychologists for stigma reduction is to improve the quality of mental health services. It can be useful to consider workshops and in-service training course for healthcare providers in this regard. A stakeholder mentioned:

*“For overcoming stigma, specialists and even experienced physicians and psychiatrists should be retrained.”* (P_5_FGD_2_)

#### Public education

All of the participants believed that public education, professional education, and providing a course in the field of mental health for all non-medical students are effective factors for stigma reduction. Providing structured psycho-education for the families of patients via different methods, emphasizing on the biological etiology of mental disorders, may lead to stigma reduction. A participant said:

*“I want to focus on this issue that when we face with a mental disorder in our families, everyone gets confused. I wish we were aware previously through the media or at least get information through textbooks or compulsory courses at universities.”* (P_8_FGD_1,_ SSI)

Children’s attitude can be guided in early ages by providing pre-school and school programs and inclusion of life skills as subjects in the educational curriculum. It can be useful to teach students facing a patient with mental disorder and to include the mental health literacy in educational programs. A participant emphasized:

*“Social awareness is at the top of all solutions. It can be started from very childhood or be included in the textbooks by the Ministry of Education”.* (P_8_FGD_2_)

#### Utilizing the potential of Islamic missionaries

Some stakeholders believed that the role of Islamic missionaries is undeniable to everyone in Iran. It can be considered as a privilege in reducing stigma. Seminaries, Islamic Development Organization, and mosques and religious ceremonies including Juma [congregational Friday] Prayer places are suitable to reduce stigma. Religious teachings and various narratives of religious leaders indicate that Muslims were pioneer in stigma reduction. A clergyman said:

*“One day the prophet of Islam [Mohammad (pbuh)] saw a kid throwing stones at a mentally ill patient. He stopped that kid and said: “He is a patient, we are not allowed to throw stones at these patients or mock them”.* (P_4_WN)

### Changing the culture

The majority of the participants mentioned that the culture of every society results from their persistent traditions, beliefs and customs. A systematic plan trying to create new culture may be beneficial for stigma reduction.

#### Establishing cultural committees, launching campaigns, and determining a cultural ambassador

One of the important issues in stigma reduction is modifying the public beliefs and culture through establishing cultural committees, advertising campaigns, and selecting an ambassador who is interested to participate in such programs. Additionally, establishing think-tank and integrated organizations is a great support in these campaigns. A psychologist said:

*“Unfortunately, some conceptions about mental patients have been established among the people. For example, we hear that some people say “he/she is faint-hearted, weak, or aimless.”* (P_7_FGD_1_)

#### The role of media

All of the participants believed that media plays an undeniable role in reducing or increasing stigma. We can use the capacity of media for proper training and modifying wrong beliefs under the supervision of specialists and experts. A psychiatrist mentioned:

*“Media is like a university. It can either generate or reduce the stigma. It can increase the psychological knowledge of people through multiple programs.”* (P_1_SSI)

Educating and training of planners and movie makers could be beneficial. However, implementation of these programs is associated with some difficulties, and it is a time consuming process as well. A psychiatrist said:

*“Building culture for movie producers and directors is a time consuming process. We cannot expect them to execute all psychological instructions immediately.”* (P_10_FGD_2_)

#### The role of books and educational materials

Widespread use of books and educational materials has a profound and effective role on stigma reduction. A participant mentioned:

*“Writing appropriate books is a suitable solution in reducing stigma. It can help to re-introduce mentally ill patients to the society and show that they are like normal people in many ways. Thus mental disorders will be taken like many other diseases”*. (P_8_FGD_2_)

A few participants believed that due to the expansion of social networks and their impact on the majority of people, it is expected that a significant effect will be achieved by using them. These networks can provide an appropriate educational context. A family member of mental patient said:

“*We can use social networks to promote the awareness of society by explaining the symptoms of disorders.”* (P_8_FGD_2_)

#### Holding festivals

Launching cultural, artistic or athletic festivals with diverse range of general or specific audiences to reduce stigma was emphasized by some participants. Here, we can exhibit the artistic work of patients as well as the works of others with mental disorder theme.

#### The role of popular individuals

Nowadays, celebrities and famous people including athletes, national heroes, artists, and politicians are invited to cooperate or help in many charity activities. The support of these people increases social motivation and is effective in modifying public attitudes. A recovered mentally ill person said:

*“Well-known mentally ill patients should be introduced to the public. Like the great Iranian poet who was diagnosed with bipolar disorder.”* (P_3_FGD_1_)

#### Introducing recovered patients

Some participants mentioned that highlighting recovered psychiatric patients and using their experiences in different programs like festivals, group therapy, and seminars are effective in reducing stigma. A psychologist said:

*“We know many treated patients already suffering from mental disorders for years who are now successful and well-known. Sometimes, they are not willing to be recognized, but we have to introduce them to others.”* (P_7_FGD_1_)

#### Creating a common literature

In today’s world, every discipline has a specific literature and terminology. Some participants said that due to the specific value of some words, it is required to create a common literature among the professionals in order to address the patients with commonly accepted and positive value terms, not unrelated words. A participant as a media expert said*:*

*“We have to change associations or create appropriate words to them; otherwise, we will face with difficulties. Eventually, remodeling a building has many problems in comparison with building a new one!”* (P_10_FGD_2_)

### Promoting supportive services

#### Budget and insurance coverage

Allocation of sufficient funds for stigma reduction was emphasized by all the participants. A participant mentioned:

*“They have to allocate sufficient budget. For example, artists are full of good ideas but obviously they need money! Making movies, producing a CD, and writing a book are costly. It is a good idea to give a CD to patients and their families, but this also needs budget!”* (P_8_FGD_1_)

Some participants believed that even though the burden of mental disorders is more than that of physical disorders, it seems that there is discrimination in allocating budget. The majority of the participants suggested that insurance organizations become more specific and pervasive. A few participants proposed that chronic psychiatric patients should be considered as “special patients” for specific budget allocation. A board member of the Support Forum of Schizophrenia Patients said:

*“Psychiatric patients, especially schizophrenic ones should be considered as special patients to be able to use insurance subsidies.*” (P_8_FGD_2_)

#### Necessity of defining appropriate tariffs for mental health services

The majority of the participants said low tariffs for mental health services and stigmatization are correlated to each other. They emphasized that these services should be valued proportional to other medical services, and even more attention should be paid to mental health services to avoid discrimination among different disciplines. A psychiatrist said:

*“We have to consider a special position for psychiatrists and clinical psychologists. We need experts to allocate tariffs for them.”* (P_1_FGD_2_, SSI)

#### Consideration of the social rights for patients

Providing better facilities for psychiatric patients indicates that they have similar rights like other citizens. Due to the fact that these patients require having a normal life, it seems to be necessary to modify the existing structures; this will also provide social rights for the patients. A participant said:

*“Promoting the quality of life and providing facilities for mental patients enhance their dignity in the society. These actions represent that mental patients have similar rights like the normal people. … Subsequently, the society would have a more positive attitude toward them.”* (P_11_WN)

Organizations’ support increases the patients’ cooperation and acceptance. Also establishing special centers and utilizing the potential of psychiatrists, psychologists, occupational therapists, social workers, and psychiatric nurses can be helpful to increase acceptance in patients and their families.

### The role of various organizations and institutions

#### Ministry of Health and Medical Education

As a custodian of mental health, the MoHME should be powerful, and as the first step, it should strengthen the Mental Health Office. Some participants said that the mental health structure should be promoted and play a crucial role. A psychiatrist said:

*“Certainly, the MoHME cannot be alone. It is necessary that other organizations be in accordance with its policies.”* (P_1_FGD_1_)

#### Municipality

It is important to reduce stigma toward mental disorders using the potential of the municipality and its communications with different districts and health centers. Municipality has a corporate status and power of self-government or jurisdiction. A representative of the municipality said:

*“We communicate with all citizens. Indeed, our target population is not a special group; we can utilize the potential of health centers to educate people. … We can broadcast educational messages through the subways monitors. Also we can cooperate to produce different educational materials.”* (P_5_FGD_2_)

#### Islamic Development Organization (IDO)

IDO was established with the aim of propagation the culture of real Islam, manifesting the spiritual life, and disseminating the belief and faith values. Some participants emphasized on very important role of clergymen in cities, towns, and even remote villages in stigma reduction. Other effective religious bodies are the Soureh University, Soureh Movie Makers Group, etc. A clergyman mentioned:

*“Clergymen in different cities are prepared to do advertising in the field of stigma reduction in mental issues if they have a clear-cut programmed comprehensive plan.*” (P_4_WN)

#### Other organizations

Ministry of Culture and Islamic Guidance; Ministry of Science, Research and Technology; Ministry of Education; Iranian Health Education and Promotion Association; and Welfare Organization can play a major role in stigma reduction in terms of education, publications, and providing internships and job opportunities. In addition, using the potential of non-governmental organizations (NGOs), military organizations, kindergartens, mosques, seminars, telecommunication, Friday Prayer Imams, poets, writers, Institute for the Intellectual Development of Children and Young Adults, The Children’s Book Council, and the Parliament were emphasized by the majority of the participants. There are few NGOs (such as AHEBA: an Iranian NGO for supporting schizophrenia patients) giving services to mental disorder patients, or they do not have an executive authority in stigma reduction in Iran. The chief of the Support Forum of Schizophrenia Patients (AHEBA) said:

*“There are very few NGOs active in mental disorder issues, or do not have enough power. We must empower them.”* (P_2_FGD_1_, SSI)

The MoHME should identify the potentials of different organizations, and then direct and make best use of them in reducing stigma.

### Integrated reform of structures and policies

#### Establishing committee and secretariat

Establishing a committee in accordance with the World Psychiatric Association (WPA) programs to collect data and organize a strategic council is of priorities in stigma reduction. This committee should have a special structure that can coordinate professional and occupational committees to support patients. Some participants emphasized that presence of secretariat and team work is necessary to avoid parallel efforts and costs. A psychiatrist mentioned:

“*We need establishing a committee for data collection about this phenomenon; then we do not need big decisions*.” (P_9_FGD_1_)

#### S*etting professional boundaries and preventing the involvement of non-experts*

It seems that demarcation between different disciplines and preventing the involvement of non-experts is necessary. To have a consistent routine, we have to define roles and positions. A participant said:

“*There are no clear boundaries between therapeutic structures in Iran. … It means that we couldn’t define boundaries. The organizations should define delimitations so that non-experts are not allowed to interfere in the diagnosis or treatment process. It rarely happens in medicine that non-expert of a special field interferes in the treatment course of a heart disease or cancer, but in the field of mental disorders, such intervention of non-experts is very common.”* (P_1_FGD_2_)

#### Integration of psychiatric wards in general hospitals

Some participants emphasized that establishment of psychiatric units within general hospitals and launching psychosomatic wards in general hospitals are important activities to reduce stigma. Allocation of 10% of the general hospitals’ beds to mental patients was advised. A psychiatrist mentioned:

“S*ix years ago, we have to set up psychosomatic and psychiatric wards in general hospitals. In this way, patients will refer more easily, and also stigma will be reduced*”. (P_1_FGD_2_)

#### Emphasis on having systematic and comprehensive programs

Some participants mentioned that having formulated and planned programs can help to reduce stigma in both basic and specialized levels. It requires coherent and detailed programs along with direct involvement of the relevant institutions. Through making accurate policies and unity, we can reduce stigma and avoid parallel works and duplications. A participant said:

*“We have to make proper policies, and other organizations and institutions should comply with it. We should not be separated and dispersed”.* (P_5_FGD_2_)”

### Evidence-based actions

Some participants believed that research is the cornerstone of any great executive action. Certainly, based on research results, through evidenced-based plans, we can find an effective strategy to reduce stigma.

#### Research actions

The transformation of requirements into data and statistics leads to an accurate plan. In this regard, we have to evaluate available studies and information. It is clear that first, needs assessment should be conducted. A professor of the University of Social Welfare and Rehabilitation Sciences said:

*“We hope to find a clear statistical language to provide solutions to the related organizations through research-based methods”.* (P_9_FGD_2_)

#### Using successful projects as a pattern

We can implement successful programs using the experience of successful models implemented in other parts of the world, the opportunity of collaboration with the UNESCO in Iran that works actively on the field of mental health, and HIV/AIDS and addiction stigma reduction programs as sources of inspiration and benchmarking.

## Discussion

Data analysis resulted in six main themes. The most important strategy in the present study was promoting awareness of people. One main action is providing education that leads to change stereotype beliefs among different folks of people, therapists, policy makers, media professionals, and patients and their families. These educations should be research-based and adopted with cultural needs, especially taking the target groups like adolescents. In this regard, Mansouri et al. found that education was effective on modification of attitudes and promoting knowledge [30]. Other studies pinpointed the role of promoting education and general knowledge toward psychiatry [31–33].

In this study, the findings showed that there was stigma and discrimination among the medical staff including physicians, other specialists, and even psychiatrists. The participants emphasized on changing knowledge and attitudes of these groups about stigma. According to Adewuya and Oguntade, about half of the physicians believed that the pathology of mental disorder pertains to supernatural powers, and that mentally ill patients are dangerous, unpredictable, aggressive, and out of control. Majority of them (61.4%) agreed with maintaining social distance with psychiatric patients. They also believed that the possibility of recovery in mental patients is poor [34]. In another study, the attitudes of nurses in five different European countries were completely different. Portuguese nurses had positive view and Lithuanian nurses had negative view [9]. The participants in the present work were hopeful about changing attitudes and beliefs among students through proper education from the first years of their medical course. Obviously, educational programs for this group of people would be more scientific and accessible. The present study showed that medical students achieve their information about mental patients through their previous knowledge and media, and not from scientific literature and contact with patients. A study revealed that 30% of the students agreed that the most important way to get information about mental disorder is watching movies [35]. Various studies have indicated that there is a negative attitude among students toward mental patients [36–38]. Although few researches have reported that appropriate psychiatric training has positive effect on the attitudes of medical students [36], it has also been revealed that clinical internship courses in psychiatric wards is an effective factor in changing the attitudes of students [39, 40]. Evidence shows that interns have more positive attitude toward psychiatric patients in comparison with other medical students [41]. Therefore, to reduce stigma among medical professionals, providing a training program in educational curriculum is highly recommended [42].

According to the participants’ view, a practical and important advice for medical students is regular presence in psychiatric wards along with precise supervision. Similar findings have been reported by other researchers [39].

The participants emphasized on the cultural and social contexts in Iran. Stigmatizing attitudes and fear from mentally ill patients have roots in culture [16]. Due to the important role of clergymen in different aspects of Iranian society, the participants agreed that their beliefs and opinions could be effective in shaping the attitudes of people. Therefore, it was emphasized to have joint programs with this influential and powerful group. IDO with regard to possession of nearly 2000 missionaries in various parts of the country has a high potential to contribute to stigma reducing programs. The culture of every society is resulted from their persistent traditions, beliefs and customs. Our participants mentioned that in Islam’s point of view on mental disorders is related to providence, it is not guilt or God’s torment. Therefore, this view should be emphasized and described by the clergymen to the different folks of people. Also despite the similarities reported in other studies [17], attention to the various ethnic groups of people in Iran is strongly suggested.

Another strategy for stigma reduction was to change the available treatment structures. The new structures give more importance to mental patients by meeting their financial needs and giving them better insurance coverage, paying due attention to the needs of therapists, the relationship among the members of therapeutic team, and therapeutic alliance. Aviram also emphasized on improving the interpersonal relationship between patients and therapists [43]. Meanwhile, in a similar article, it has been mentioned that good insurance coverage has a crucial role in stigma reduction [17].

From years ago, socializing psychiatry and its exclusion from psychiatric hospitals was emphasized as an important step in reducing stigma. In this study, the participants emphasized on revising the treatment structures and removing the existing defects. Similar findings have been reported in other studies [44].

The participants considered the following actions as the most important steps in reducing stigma in the country: integration of mental health services in other health services and establishment of psychiatric units within the general hospitals. This finding has been confirmed in other studies as well [45–47].

The stakeholders believed that stigma is widespread, and hoped that, like many other countries, the stigma reduction programs can attract the cooperation of governmental organizations, social activists, and NGOs. The findings showed that NGOs do not have an executive authority in stigma reduction in Iran. However, other Iranian NGOs that allocate their potentials to diseases such as cancer and other chronic illnesses have shown more success. This finding is in line with the study of Hanafiah that emphasized on the essential role of NGOs in stigma reduction [17].

Our findings showed that a stigma reduction program should be a constant and continuous process, and it should not be considered only for certain days or occasions. Also cultural and artistic efforts can be very helpful in this regard. Moreover, some temporary campaigns can be launched to attract the attention of people.

This study showed that many other organizations and centers can help in the design and implementation of stigma reduction programs. At a first glance, it seems better to integrate all these organizations under the provision of a custodian. Therefore, considering a committee or office for coordination of various programs is essential.

The participants believed that there is a systematic discrimination in budget allocation. Even though the burden of mental disorders is much more than physical illnesses [48], there is an evident discrimination in allocating budget among the policy makers. This finding is similar to other studies [44, 49, 50].

The current study revealed that the media in Iran illustrates a negative image about psychiatrists and psychiatry. Previous attitudes and background of movie-makers and producers have a prominent role in their products. Moreover, the participants believed that the majority of information in the field of mental health is misleading. These findings were confirmed in other studies [51, 52]. Therefore, actions should be taken to change such beliefs and attitudes. The participants stated that it is necessary to utilize the views of psychiatrists and psychologists in the media to reduce stigma. Other studies are in line with this study. When negative news is spread by the media, negative attitudes will form and spread quickly [53].

The participants hoped that successful educational programs would lead to a common literature and terminology. Furthermore, a precise concept about psychiatry should be provided for medical students, and positive role models should be introduced [54]. Unfortunately, in Iran, due to the negative value of some words, we need to create a common literature among the professionals; so, patients would not be addressed with negative words. Also we should not use the word “spirit” because it is related to religion; so, a procedural unity will be created in using words.

The findings of the present study emphasized to the use of evidence-based guidelines to decrease stigma. Research–based actions, needs assessments based on statistics, and using successful projects as a pattern can be starting points for stigma reduction. Other studies have reported similar findings [44].

Finally, it seems that now we have suitable conditions for implementation of the participants’ proposed solutions in Iran.

### Limitation

One limitation of the present research was the qualitative nature of the study. The small number of participants in this study was other limitation, as the qualitative nature of the research does not allow the data to be generalized. Another limitation of the study was that the topic had scanty of literature for review. Further research using qualitative and quantitative approaches is needed to evaluate the impact of these strategies in the actual situation. Moreover, we were unable to assess the effectiveness of emerging strategies due to time limitation.

## Conclusion

In summary, the present study aimed to explore an exhaustive perspective about the stigma reducing strategies used by different stakeholders, patients and their families. Some strategies were emphasized more than others by the participants. Emphasis on education and changing the attitudes and culture emerged as key factors to reduce stigma. It is recommended to conduct further research for assessment of the reduction strategies of stigma toward people with mental disorders in other settings.

## Abbreviations

FGD: Focused group discussion
IDO: Islamic Development Organization
MoHME: Ministry of Health and Medical Education
NGO: Non-Governmental Organizations
P: Participant
SSI: Semi-structure interview
WHO: World Health Organization
WN: Written narrative
